# Back to the future: what would the post-2015 global development goals look like if we replicated methods used to construct the Millennium Development Goals?

**DOI:** 10.1186/1744-8603-10-19

**Published:** 2014-04-03

**Authors:** Claire E Brolan, Scott Lee, David Kim, Peter S Hill

**Affiliations:** 1 School of Population Health, The University of Queensland, Herston Road, Herston, Brisbane, Queensland 4006, Australia

**Keywords:** Millennium Development Goals, Global health policy, Post-2015 development agenda

## Abstract

**Background:**

The Millennium Development Goals (MDGs) were ‘top-down’ goals formulated by policy elites drawing from targets within United Nations (UN) summits and conferences in the 1990s. Contemporary processes shaping the new post-2015 development agenda are more collaborative and participatory, markedly different to the pre-MDG era. This study examines what would the outcome be if a methodology similar to that used for the MDGs were applied to the formulation of the post-2015 development goals (Post-2015DGs), identifying those targets arising from UN summits and conferences since the declaration of the MDGs, and aggregating them into goals.

**Methods:**

The UN Department of Economic and Social Affairs (DESA) list of major UN summits and conferences from 2001 to 2012 was utilised to examine targets. The DESA list was chosen due to the agency’s core mission to promote development for all. Targets meeting MDG criteria of clarity, conciseness and measurability were selected and clustered into broad goals based on processes outlined by Hulme and Vandemoortele. The Post-2015DGs that were identified were formatted into language congruent with the MDGs to assist in the comparative analysis, and then further compared to the 12 illustrative goals offered by the UN High-Level Panel of Eminent Persons on the Post-2015 Development (High-Level Panel) Agenda’s May 2013 report.

**Results:**

Ten Post-2015DGs were identified. Six goals expressly overlapped with the current MDGs and four new goals were identified. Health featured prominently in the MDG agenda, and continues to feature strongly in four of the 10 Post-2015DGs. However the Post-2015DGs reposition health within umbrella agendas relating to women, children and the ageing. Six of the 10 Post-2015DGs incorporate the right to health agenda, emphasising both the standing and interconnection of the health agenda in DESA’s summits and conferences under review. Two Post-2015DGs have been extended into six separate goals by the High-Level Panel, and it is these goals that are clearly linked to sustainable development diaspora.

**Conclusions:**

This study exposes the evolving political agendas underplaying the current post-2015 process, as targets from DESA’s 22 major UN summits and conferences from 2001 to 2012 are not wholly mirrored in the HLP’s 12 goals.

## Background

In 2001, the year following the release of the United Nations (UN) Millennium Declaration, a small group of UN experts with colleagues from the Organisation for Economic Cooperation and Development’s Development Assistance Committee (OECD/DAC), the World Bank, and the International Monetary Fund (IMF), systematically worked through the targets included in the Millennium Declaration, drawn from the previous decade of UN Summits and Conferences, identifying those targets that met the criteria of clarity, conciseness, and measurability [1,2]. The eighteen targets selected were clustered into the eight Millennium Development Goals (MDGs), and included in the *Road map towards the implementation of the United Nations Millennium Declaration*[3], presented to the UN by Secretary-General Kofi Annan on 6 September 2001, and endorsed the next year at the International Conference on Financing for Development at Monterrey [4]. The goals gained international credibility and have facilitated laudable poverty-reduction outcomes [5,6], despite some early political resistance [7,8], and less promising progress for some goals—in particular MDG 5 (Improving Maternal Health) [9-11].

Against this backdrop the international community is now formulating a new human development agenda to improve the lives of the world’s growing population of 7 billion. The contemporary policy landscape in which this occurs differs significantly to the context surrounding the MDGs’ creation. The current UN-led process is far more collaborative and participatory, as evidenced by the UN’s 11 thematic consultations [12,13]. The global development community has also learnt important lessons from MDG implementation, including how their distortion prioritised certain agendas [14]. Of greatest significance, however, is the fact the post-2015 development goals (Post-2015DGs) are being configured in a dynamic global landscape that is markedly different from that which framed the pre-MDG era. Key influencing factors include: the impact of the Global Financial Crisis since 2008; the economic shift of many populous countries into middle income status; recognition that the bulk of the world’s poor are now found in middle and not low income countries; the ubiquitous nature of new communication technologies; the rise of the climate change agenda; and the complex global governance systems that have evolved partly in response to the MDGs [15]. The parallel, State-driven Sustainable Development Goal (SDG) agenda adds further dimension to, and influence on, post-MDG debate; effectively extending the parameters of the debate in terms of ecological goals, concepts of planetary boundaries and resilience.

Although concern exists the MDGs were formulated in a paternalistic prism by policy elites with usual geopolitics predominating [16-18], the methodology of identifying the MDGs from goals set in UN summits and conferences in the 1990s is not without its own validity. Vandemoortele [2] argues debate in the 1990s both within and following the UN Summits and Conferences allowed for consultation and consensus, which implicitly legitimized the selection of the MDGs, and subsequent endorsement of the Secretary-General’s Road Map.

While the current process of proposing the Post-2015DGs is explicit in its consultation—through national, thematic and individual processes—this research aims to provide an alternative benchmark, against which the development of the Post-2015DGs might be compared. The analysis examines what would the outcome be if a methodology similar to that used for the MDGs were applied to the development of the Post-2015DGs, identifying those targets arising from the UN summits and conferences since the declaration of the MDGs, and aggregating them into goals. These findings will then be further compared to the 12 illustrative goals offered by the High-Level Panel of Eminent Persons on the Post-2015 Development Agenda (High-Level Panel) in its May 2013 report.

## Methods

A literature review was performed on the formulation of the MDGs. An in-depth interview was also conducted by PSH with Jan Vandermoortele on 20 August 2012 in Bruges, Belgium. Jan Vandermoortele has written extensively on MDG implementation and was intimately involved in the MDG architecture through his role as Director of the Poverty Group at UN Development Program in New York from 2001 – 2005.

In order to identify the UN sponsored summits and conferences for analyses in this research following the declaration of the MDGs in 2001, we utilised the UN Department of Economic and Social Affairs (DESA) list of Major UN Summits and Conferences between the period of 2001 to 2012 (Table 1). The DESA list of Major UN Summits and Conferences was chosen in view of the agency’s core mission to promote development for all. DESA identified twenty-two UN events in this time period.

**Table 1:** DESA’s List of Major UN Conferences and Summits 2001 – 2012

**Year**	**UN event**	**Location & date**
**2001**	Third UN Conference on the Least Developed Countries	Brussels, 14–20 May
	Implementation of the outcome of the UN Conference on	New York, 6–8 June
	Human Settlements (Habitat II)	*Special Session of the GA
	Problem of human immunodeficiency virus/acquired immunodeficiency syndrome (HIV/AIDS) in all its aspects	New York, 25–27 June
		*Special Session of the GA
**2002**	International Conference on Financing for Development	Monterrey, 18–22 March
	Second World Assembly on Ageing	Madrid, 8–12 April
	GA Special Session on Children	New York, 8–10 May
	World Food Summit: five years later	Rome, 10–13 June
	The World Summit on Sustainable Development	Johannesburg, 26 August–4 September
**2003**	International Ministerial Conference of Landlocked and Transit Developing Countries and Donor Countries and International Financial and Development Institutions on Transit Transport Cooperation	Almaty, 28–29 August
	The World Summit on the Information Society	Geneva, 10–12 December
**2005**	World Conference on Disaster Reduction [Round-up]	Kobe, 18–22 January
	10-year review of the implementation of the Copenhagen Declaration and Programme of Action and the outcome of the 24th special session of the General Assembly	New York, 9–18 February
	Beijing +10 Conference: Forty-Ninth Session of the Commission of the Status of Women	New York, 28 February–11 March
	The 2005 World Summit - High-Level Plenary Meeting of the 60th Session of the General Assembly	New York, 14–16 September
	The World Summit on the Information Society – Second phase	Tunis, 16–18 November
**2006**	High Level Dialogue on International Migration & Development	New York, 14–15 September
**2008**	High Level Event on the Millennium Development Goals	New York, 25 September
	Follow-up International Conference on Financing for Development to Review the Implementation of the Monterrey Consensus	Doha, 29 November–2 December
**2009**	United Nations Conference at the Highest Level on the World Financial and Economic Crisis and Its Impact on Development	New York, 24–26 June
**2010**	Summit on the Millennium Development Goals	New York, 20–22 September
**2011**	Fourth United Nations Conference on the Least Developed Countries	Istanbul, 9–13 May
**2012**	Rio + 20: United Nations Conference on Sustainable Development	Rio de Janeiro, 20–22 June

SL and DK performed the primary analysis of the twenty-two UN summits and conferences, identifying targets from the key documents released from the summits or conferences, which included Conference or Outcome Reports, Final or Summary Documents, and Summit Declarations. Targets that met the MDG criteria of clarity, conciseness and measurability were selected and clustered into broad goals based on the processes outlined by Hulme and Vandemoortele [1,2]. The Post-2015DGs that were identified were then formatted into language congruent with the MDGs to assist in our comparative analysis.

For inter-rata reliability, CB then performed a second round of analysis of DESA’s twenty-two UN summits and conferences from 2001 to 2012, and synthesised emerging targets and goals with those initially developed by SL and DK. CB and PH cross-checked the data to confirm research findings. Comparisons of the goals derived by this process were then made with the MDGs and those goals recently proposed by the High-Level Panel.

## Results

From analysing the targets arising in twenty-two DESA selected major UN summits and conferences from 2001 to 2012, ten Post-2015DGs were identified and named utilising comparative MDG language (Table 2).

**Table 2:** Comparing the MDGS with this study’s findings

**8 MDGs**	**10 Post-2015DGs**
1. Eradicate Poverty	1. Eradicate Poverty
2. Universal Primary Education	2. Universal Primary Education of Good Quality
3. Promote Gender Equality	3. Ensure Gender Equality, Women’s Empowerment, Health and Well-Being
4. Reduce Child Mortality	4. Protect Children’s Lives and Rights
5. Improve Maternal Health	5. Optimise the Health and Well-Being of the Ageing
6. Combat HIV/AIDs, Malaria and Other Diseases	6. Combat HIV/AIDs, Tuberculosis, Malaria and Other Communicable Diseases
	7. Ensure Food and Water Security
7. Ensure Environmental Sustainability	8. Ensure Sustainable Development
	9. Create Universal Access to Communication and Information
8. Global Partnership for Development	10.Global Partnerships in Governance and Financing for Development

We identified six goals that expressly overlapped with the current MDGs relating to poverty, education, gender, HIV/AIDs and other communicable diseases, environmental sustainability, and global partnership for development. The title of five of these goals was reworked to reflect new and emerging language (and, importantly, priorities) within the UN summits and conferences from 2001 onward: “MDG 2 - Universal Primary Education” became “Post-2015DG 2 Universal Primary Education of Good Quality” to reflect emphasis within the relevant UN summit or conference on not only ensuring children globally complete primary education, but that all children also receive an education of quality standard; “MDG 3 – Promote Gender Equality” expanded into “Post-2015DG 3 Ensure Gender Equality and Women’s Empowerment, Health and Well-being” to highlight the broader and more forceful language within the relevant summits and conferences on not solely “promoting” but “ensuring” the equality and empowerment of the world’s women, with particular emphasis on meeting their health needs; “MDG 6 – Combat HIV/AIDs, Malaria and Other Diseases” became “Post-2015DG 6 – Combat HIV/AIDs, Tuberculosis, Malaria and Other Communicable Diseases” so as to incorporate prominence of tuberculosis within the summits and conferences analysed; “MDG 7 – Ensure Environmental Sustainability” became “Post-2015DG 8 - Ensure Sustainable Development” to reflect the UN’s consistent and integrated focus on the sustainable development agenda (that included environmental sustainability); and “MDG 8 – Global Partnerships for Development” became “Post-2015DG 10 – Global Partnerships in Governance and Financing for Development” to encapsulate UN emphasis on the relationship between governance and financing for development, which gained prominence in response to the 2008 Global Financial Crisis. While poverty was not expressly addressed in the titles of DESA’s major summits or conference between 2001 and 2012, both the explicit and implicit pervasiveness of poverty eradication as an overarching goal to be generated from the achievement of other inter-related agendas necessitated locating poverty eradication as Post-2015DG 1.

We also identified four new goals, distinct from the MDGs: “Post-2015DG 4 – Protect Children’s Lives and Rights”; “Post-2015DG 5 – Optimising the health and wellbeing of the Ageing”; “Post-2015DG 7 – Ensure food and water security”; and “Post-2015DG 9 – Create universal access to information and communication”. The broad inclusion of children in the outcome documents and targets of the UN conferences and summits under analysis, combined with the GA’s Special Session on Children in 2002, warranted creation of a separate children’s goal within the Post-2015DGs. To ensure consistency with Post-2015DG 4, we included a separate women’s goal (Post-2015DG 3) but also extended the title to incorporate health and well-being factors to reflect prominence of women’s health issues in the content of the summits and conferences analysed. Therefore MDG 5 on Maternal Health is included within Post-2015DG 3. Of further note, the new goal on ageing (Post-2015DG 5) would also incorporate the non-communicable disease (NCD) and shelter agendas that also received status in the content of the DESA summits and conferences under review.

Health featured prominently in the MDG agenda, with three explicit health goals within the eight goals, and continues to feature strongly in four of our ten Post-2015DGs. However, our Post-2015DGs reposition health within umbrella agendas relating to women, children and the ageing. HIV/AIDs notably maintains its prominence, together with tuberculosis, malaria and other communicable disease in Post-2015DG 6. The underlying determinants of health highlighted by the UN Committee on Economic, Social and Cultural Rights (CESR) in its General Comment No. 14 on the Right Highest Attainable Standard of Health (2000), including “food and nutrition, housing, access to safe and potable water and adequate sanitation, safe and healthy working conditions, and a healthy environment”, are also encapsulated within Post-2015DGs on children, women and the ageing. However the underlying determinants are also evident within Post-2015DG 6 (HIV/AIDs, tuberculosis, malaria and other communicable diseases), Post-2015DG 7 (food and water security) and Post-2015DG 8 (sustainable development). Therefore six of the 10 Post-2015DGs incorporate the right to health agenda, emphasising both the standing and interconnection of the health agenda in DESA’s summits and conferences just beyond first decade of the 21st century.

In comparison with the High-Level Panel’s 12 goals (Table 3), there is also consistency with the content of our 10 Post-2015DGs, notably on poverty and Post-2015DG 2’s emphasis on quality education. The High-Level Panel similarly features a specific women’s goal, but expressly extends this to include girls, affirmatively seeking to “empower” and “achieve” the inherent rights of women and girls, opposed to the arguably more protectionist position (to “ensure”) articulated by Post-2015DG 3. In addition, the four targets and indicators under the High-Level Panel’s Women and Girls goal (HLP-G 2) do not include an express health focus like Post-2015DG 3.

**Table 3:** Comparing MDGs, Post-2015DGs with the High-Level Panel’s (HLP-Gs) 12 illustrative goals

**8 MDGs**	**10 Post-2015DGs**	**12 HLP-Gs**
1. Eradicate Poverty	1. Eradicate Poverty	1. End Poverty
2. Universal Primary Education	2. Universal Primary Education of Good Quality	3. Provide Quality Education and Lifelong Learning
3. Promote Gender Equality	3. Ensure Gender Equality, Women’s Empowerment, Health and Well-Being	2. Empower Girls and Women and Achieve Gender Equality
4. Reduce Child Mortality	4. Protect Children’s Lives and Rights	4. Ensure Healthy Lives
5. Improve Maternal Health	5. Optimise the Health and Well-Being of the Ageing	
6. Combat HIV/AIDs, Malaria and Other Diseases	6. Combat HIV/AIDs and Other Communicable Diseases	
	7. Ensure Food and Water Security	5. Ensure Food Security & Good Nutrition
		6. Achieve Universal Access to Water and Sanitation
7. Ensure Environmental Sustainability	8. Ensure Sustainable Development	7. Secure Sustainable Energy
		8.Create Jobs, Sustainable Livelihoods, and Equitable Growth
	9. Create Universal Access to Communication and Information	9. Manage Natural Resource Assets Sustainably
8. Global Partnership for Development	10. Global Partnerships in Governance and Financing for Development	10. Ensure Good Governance and Effective Institutions
		11. Ensure Stable and Peaceful Societies
		12. Create a Global Enabling Environment and Catalyse Long-Term Finance

It follows that within the High-Level Panel’s goals, health receives its own goal (HLP-G 4: Ensure Healthy Lives), which references MDGs 4, 5 and 6 in its targets and indicators, but also extends these to incorporate priority NCDs and neglected tropical diseases. Here, there is consistency with our own findings: While NCDs were not included in the MDG agenda, their emerging prevalence in the summits and conferences under review signalled their inclusion as a target or indicator under our Post-2015DG 5 on optimising the health and well-being of the ageing. Furthermore, and in comparison, our Post-2015DG 6 continued the MDG’s prominence of the HIV/AIDs and malaria agenda, but unlike the MDG agenda and similarly to the High-Level Panel’s goals, we did not identify child mortality and women’s health as distinct and separate goals. This is an interesting result given it is unlikely either of these goals will be achieved by 2015, and are the subsequent focus of in-country acceleration programs engaging UN focus and resource.

The lack of explicit focus on ageing populations within the goals, targets and indicators by the High-Level Panel contrasts with the particular prominence of this group in DESA’s major summits and conferences under review. Alternatively, the strong children’s rights agenda at play in the summits and conferences analysed is reflected within the widespread children’s rights agenda within the targets and indicators of the High-Level Panel’s 12 goals (for example, 2(a)-(b), 3(a)-(c), 4(a)-(b), 5(b), 6(a), and 11(a)).

Another dichotomy between the High-Level Panels 12 goals and our 10 Post-2015DGs is Post-2015DG 9’s emphasis on creating universal access to communication and information. This focus is also lacking within the MDGs, although Target 8(f) on making available benefits of new technologies, especially information and communications, may be one intersection. There are also intersections within the targets and indicators within the High-Level Panel’s goals, including 8(c), 10(d), and 12(f), though the elevated focus that is incumbent within a goal is lacking. This again demonstrates that the agendas of prominence within the summits and conferences in our analysis have not gained the profile they may have had utilising the methodology involved in MDG formulation.

Conversely, the second half of the High-Level Panel’s 12 goals (that is, Goal 7 through to Goal 12), both emphasises and unpacks our Post-2015DGs 8 (sustainable development) and 10 (global partnership). Indeed, there is interface in target content between our Post-2015DG 8 and the High-Level Panel’s Goals 7 to 9, as well as our Post-2015DG 10 and the High Level Panel’s Goals 10 to 12. This finding highlights the constraints of a more reductionist approach taken by MDG formulators, and replicated in our study.

## Discussion

By using the methodology that underscored MDG formulation, we identified 10 Post-2015DGs to frame the post-2015 development agenda. Of our 10 Post-2015DGs, six goals replicated the MDG agenda (poverty, education, gender, HIV/AIDs and other communicable diseases, sustainability, and global partnership for development). We identified four new goals distinct to the MDGs, two of which together with the gender goal place more prominence in our Post-2015DGs than within the MDGs (and High-Level Panel goals) on the holistic needs and rights specifically of the world’s women, children and ageing populations. Elevation of the women and children’s rights agendas within the Post-2015DGs alternatively highlights the loss of MDG status of maternal and child health, which becomes subsumed as either a target or indicator within the broader goal. This is interesting given concern around the unlikely achievement of MDG 4 and 5 by December 2015.

Our one new goal on food and water security (Post-2015DG 7) is consistent with the High-Level Panel’s two separate food and water goals, again distinct from the MDGs. Post-2015DG 9 on universal access to information and communication is neither an express goal contained with the MDGs nor High-Level Panel’s 12 goals, although implicitly evident within some of the targets and indicators that fall under the High-Level Panel’s goals. While the High-Level Panel express one explicit health goal to “Ensure Healthy Lives”, Post-2015DG 6 perpetuates elevation of MDG 6 on HIV/AIDS and other communicable diseases in the post-2015 process. While our 10 Post-2015DGs do not have an all-encompassing, broad health goal like the High-Level Panel’s fourth goal, nevertheless a strong health agenda permeates our Post-2015DGs, greater than within the MDGs and the 12 High-Level Panel goals.

In contrast to the Post-2015DG’s two goals on sustainable development and global partnership, the High-Level Panel place greater weight on the issues contained within these agendas (global security, peace, governance, and the world’s fiscal and sustainable development agenda) through expanding these two goals into six. This is an enormous shift from the MDG agenda; unsurprising in light of the very different landscape the contemporary world finds itself in as it experiences the aftershocks of the Global Financial Crisis of 2008, as well as the gathering momentum around environmental and sustainable development through the State-led Open Working Group process gaining pace after the Rio + 20 Conference in June 2012.

Our analysis has produced ten goals that, just over a decade on, largely build on the substance of the MDGs. The General Assembly meeting in September 2013 will reiterate that emerging agendas must be prefaced with the fact the MDGs are not yet achieved. Indeed an accurate assessment of MDG progress will be unavailable until at least two years after 2015 to allow time for data to be processed and analysed. Hence there is growing anxiety that prematurely increasing the number of goals will only undermine their manageability and lead to loss of focus not only on the new goals, but finalising achievement of the ‘old’ ones. Bill Gates, for example, has recommended continuity with current goals but with a recalibration of targets [19,20].

One factor is clear, however, that the ‘second phase’ of the post-2015 debate will play out in the hands of the UN member-states as they involve themselves in the parallel SDG agenda, with the September 2014 release of the Open-Working Groups’ report providing an important signal on the post-2015 road. In this process, states can consider the content of the UN thematic consultations in their deliberations but are not bound to. Interestingly, the SDG process and its intersection with formulation of the UN-led post-2015 agenda parallels the dual or “twin-track” process that similarly evolved in the mid-1990s between the UN and the OECD [1]. This followed from the OECD-DAC’s formulation of seven International Development Goals (IDGs) launched in its May 1996 report (reviewing UN summit agreements) and subsequently endorsed in OECD ministerial meetings and by the G7. However in 1997 the UN took a narrower rights approach to the human development agenda than found within the IDGs. This alternate approach reflected the dynamics, constraints and interests at play as both organizations attempted to placate their different constituencies and target audiences.

Hulme [1] states the “twin track process” fuelled by separate UN and OECD lists led “to the possibility of two different sets of global poverty reduction goals in March 2001”, which is a similar risk in the contemporary global-policy landscape. In the late 1990s this possibility was reinforced by the release of two development reports in 2000 with distinct future agendas. Notably, the UN authors one of the reports (*We the Peoples: the Role of the United Nations in the 21st Century*) while the other report (*A Better World for All: Progress towards the international development goals*) was co-authored by the four major development multilaterals (the IMF, OECD and World Bank) *including* the UN. While Hulme describes the latter report as an “unprecedented show of solidarity”, the UN’s involvement in *both* reports illustrates the evolving and complicated dynamics that underscored global policy making at the turn of the century, and is cognisant today.

In addition to the “twin-track” process that emerged in the 1990s, Hulme [1] provides four other factors underlying complex, high-level policy processes that ensured the inclusion of multiple targets into the Millennium Declaration:

• Bilateral lobbying for the IDGs in 1997 and 1998, led by the UK’s Department for International Development (DFID);

• Intense negotiations over the summer of 2000 between UN actors and diverse networks jostling to ensure their interests were included in the Millennium Declaration, resulting in last-minute political compromise;

• Tension between the World Bank, IMF and UNDP over authority around the MDGs and national poverty reduction strategies (PRSPs), resolved in March 2001 facilitating reconciliation and ‘concordance’ between the IDGs and parallel UN goals; and

• US resistance to the MDGs in the early 2000s.

## Conclusion

Devising 10 Post-2015DGs by applying a methodology similar to that used for the MDGs exposes the evolving political agendas underplaying and driving the current post-2015 process, as targets from DESA’s 22 major UN summits and conferences from 2001 to 2012 are not wholly mirrored in the High-Level Panel’s 12 goals. Particularly revealing is that two Post-2015DGs have been extended into six separate goals by the High-Level Panel, and it is these goals that are clearly linked to SDG diaspora. This suggests the parallel State-led process is steering the UN’s approach to early goal formulation. There are two potential reasons: first, to create a sense of relevance and coherence within the 12 High-Level Panel goals that can facilitate future mergence of the two global-development processes; second, for the UN not to be seen by states as dominating or driving agendas, as lack of state-ownership was a political stone in the shoe for some nations when the MDGs were originally instigated.

Lessons have been learnt from the MDGs being determined from the top-down, and players and politics in the post-2015 global development landscape will not tolerate a repeat of the bureaucratically driven MDG process. The lack of ownership, participation, and partnership among high-level MDG policy makers and communities, civil society, the Global South, the private sector and other relevant stakeholders will not be repeated, especially when such actors are pivotal to MDG and post-2015 success [17,18]. Mac Darrow, Chief of the MDGs Section of the Office of the UN High Commissioner for Human Rights (OHCHR), contends global summitry commitments of the 1990s were alone insufficient to underscore the MDGs; “equally if not more important for progress is sustained political mobilisation and innovative use of the commitments” [21] p.57. With this in mind, Fidler’s [22] argument that global health governance is a form of open-source anarchy can be broadened to apply to the contemporary development space, as diverse State and non-State actors “access, adopt, apply, and adapt” to the source codes of development, recombining a limited range of potential goals, but in frames that have significantly different implications for global development. But this engagement of multiple actors in competing framings has the potential, over the next three years, to drive new goal formulation in directions more dynamic and innovative than the UN as an intergovernmental organization may have anticipated.

The strong commonalities and continuities between the three sets of goals are therefore unsurprising, as open source codes are built upon and repackaged into new formats reflecting the myriad of political, social and environmental interests. The value of the UN’s extensive consultation process to create the High-Level Panel report with 12 “illustrative” goals, does not simply lie in the capacity to develop a first draft of the goals themselves, but in demonstrating that an inclusive process is possible, and that an apparent consensus can be achieved. But that first step in the process has been completed, and UN moderation will have less influence in the next phase, as the Sustainable Development agenda is worked out through a member-state driven Open Working Groups process. In this process, the fracture lines implicit in the comprehensive High-Level Panel report may open, as the implications for the full range of nation-states—and other actors now engaged in the post-2015 process—become more apparent.

Our research has a number of limitations. The content of our Post-2015DGs would have differed had we not confined our analysis to DESA’s twenty-two “major” UN summits and conferences from 2001 to 2012. This is a limited number compared to the expansive list of UN summits and conferences occurring in the same period [23]. Further, DESA does not define when or why a UN event is “major” so as to be included on its list. A number of arguably key UN events were not included that again would have impacted on our findings, such as the High Level Meeting on Prevention and Control of NCDs in 2011, and the UN General Assembly session and resolution on global health and foreign policy for the promotion of Universal Health Coverage (UHC) in December 2012 [24]. Indeed, it is interesting UHC does not feature in our findings; paralleling UHC’s similar omission as an explicit goal or target within the High-Level Panel’s illustrative goals (and health goal in particular). We concede the lack of attention or inclusion of UHC in our own results is surprising: UHC remains highly relevant in evolving post-2015 discussion, receiving considerable support among Member States, the World Health Organization (WHO) and other key actors, emphasising UHC should in no way be ruled out as a potential post-2015 health goal or task [25-31].

Likewise, DESA’s list of Major UN Conferences and Summits completely overlooks the pivotal climate change agenda, with its antecedent links to the present post-2015 sustainable development goal agenda. For instance, the UN held a Climate Change Conference each year between 2001 – 2012, with the third World Climate Conference (WCC-3) held in Geneva in 2009. Had these key UN events been included by DESA, the content of our 10 suggested Post-2015DGs may have altered considerably.

Despite rigour in reviewing DESA data to formulate our 10 Post-2015DGs, for two reasons we remain cautious about whether replicating the MDG process can reproduce its outcomes. First, the 10 goals we propose from our research are, inherently, a cluster of goals agreed upon by the authors (we have made authorial decisions around structuring the clusters of those goals, such as locating NCDs under an ageing goal, and maternal health under a gender goal). However, given the lack of transparency and consultation in synthesising the outcomes of the UN summits and conferences of the 1990s by the close-knit-team of multilateral decision-makers, it could be argued the MDGs were, too, a cluster of goals (the content and structuring of which was) agreed upon by *those* authors. Second, our caution reflects the complex nature of policy formation that occurred in the pre-MDG period, particularly in the closed meeting rooms between the World Bank, IMF and UN at the ‘business end’ of the decision-making process. The apparent ‘simplicity’ of the MDG process conceals its highly politicized nature, and the next three years will replicate this process with more players, more politics, and more fault lines (or, rather, chasms) fracture movement toward global consensus in complex and unpredictable ways that were implicit, if not explicit, in the MDG process.

## Abbreviations

HLP-G: High-Level Panel’s Goals; IMF: International Monetary Fund; MDGs: Millennium Development Goals; OECD/DAC: Organisation for Economic Cooperation and Development’s Development Assistance Committee; OHCHR: Office of the UN High Commissioner for Human Rights; Post-2015DGs: Post-2015 development goals; SDGs: Sustainable development goals; UN: United Nations; UN DESA: UN Department of Economic and Social Affairs; UHC: Universal Health Coverage; WCC-3: World Climate Conference; OHCHR: UN High Commissioner for Human Rights; WHO: World Health Organization.

## Competing interests

The authors declare that they have no competing interest.

## Authors’ contributions

SL performed the primary analysis and first draft of findings. DK supported the primary analysis and first draft of findings. CB conducted the literature review, performed the second round of analysis and drafted the manuscript. PSH conceived the study, and participated in its design and coordination, reviewed the second round of analysis, and helped to draft the manuscript. All authors read and approved the final manuscript.
